# Histopathological Characterization and Differential Expression of miRNAs in Male Pediatric Patients With Lichen Sclerosus

**DOI:** 10.1111/andr.70157

**Published:** 2025-12-17

**Authors:** Valerie Flammang, Arndt Hartmann, Robert Stöhr, Katrin Weigelt, Carol Geppert, Frederik A. Stuebs, Matthias W. Beckmann, Bernd Wullich, Helge Taubert, Marios Marcou, Sven Wach

**Affiliations:** ^1^ Department of Urology and Pediatric Urology University Hospital Erlangen Friedrich‐Alexander‐Universität Erlangen‐Nürnberg Erlangen Germany; ^2^ Institute of Pathology University Hospital Erlangen Friedrich‐Alexander‐Universität Erlangen‐Nürnberg Erlangen Germany; ^3^ Comprehensive Cancer Center Erlangen‐EMN (CCC ER‐EMN) Friedrich‐Alexander‐Universität Erlangen‐Nürnberg Erlangen Germany; ^4^ Bavarian Cancer Research Center (BZKF) Erlangen Germany; ^5^ Central Biobank Erlangen (CeBE) Friedrich‐Alexander‐Universität Erlangen‐Nürnberg Universitätsklinikum Erlangen Germany; ^6^ Department of Gynecology and Obstetrics University Hospital Erlangen Friedrich‐Alexander University of Erlangen‐Nürnberg Erlangen Germany

**Keywords:** histopathology, inflammation, lichen sclerosus, male pediatric patients, microRNAs

## Abstract

**Background:**

Lichen sclerosus is a chronic, inflammatory, scarring disease of the skin that manifests mostly in the genital region.

**Objective:**

We studied the histomorphological characteristics, grade, and pattern of inflammation in male pediatric patients with lichen sclerosus. We also compared the expression of selected miRNAs in lichen sclerosus tissue, adjacent non‐lichen sclerosus tissue from the same patient, and healthy male pediatric patients.

**Results and Discussion:**

According to the type of inflammation/lymphocytic distribution, we categorized patients into four groups with the following features: (i) dominant lichenoid basal superficial inflammation, (ii) dominant band‐like lymphocytic infiltration in the papillary sublayer of the dermis, (iii) mixed lymphocytic inflammation combining both patterns, and (iv) lymphocytic depletion with extensive fibrosis. The extent of inflammation was graded, with patients being categorized into weak, moderate, and strong inflammation groups. In terms of miRNA expression, hsa‐miR‐146a‐5p, hsa‐miR‐146b‐5p, hsa‐miR‐150‐5p, and hsa‐miR‐155‐5p were significantly upregulated, and hsa‐miR‐199b‐5p and hsa‐miR‐200b‐3p were significantly downregulated in lichen sclerosus tissue compared with adjacent normal tissue as well as normal tissue from male pediatric non‐lichen sclerosus patients (*p* < 0.001). Hsa‐miR‐30b‐5p was significantly downregulated in lichen sclerosus patients compared with male pediatric non‐lichen sclerosus patients (*p* < 0.001). Pediatric male lichen sclerosus patients were categorized into two groups according to median age (≤9 years vs. >9 years); the early onset prepubertal patients presented, on average, a higher grade of inflammation (*p* = 0.020) and significantly higher miRNA hsa‐miR‐150‐5p (*p* = 0.049) expression compared to the older group.

**Conclusions:**

Histopathological investigations can distinguish lichen sclerosus patient groups with different extents of disease. miRNAs could serve as candidate diagnostic markers for lichen sclerosus in pediatric male patients and may represent future therapeutic targets.

## 1 Introduction

Lichen sclerosus (LS) is a chronic, inflammatory, scarring disease of the skin that manifests mostly in the genital region and can occur at any age and in both sexes. Multiple names have been used to describe the disease, such as kraurosis vulvae in female patients or balanitis xerotica obliterans in males. White spot disease, leukoplakia, and LS et atrophicus were also used until finally the term “LS” became generally accepted in 1976 [1].

LS is estimated to be highly underdiagnosed, and the exact etiology and prevalence of the disease remain largely unknown [2]. Recently, a register study reported that the incidence of LS in Sweden is 80.9 per 100,000 people per year, with a higher incidence in females (114.4) than in males (47.2) [3]. In that study, the incidence in the 0–19‐year age group was 94.5 for females and 68.6 for males per 100,000 persons between 2001 and 2020 [3].

Clinically, LS begins as white polygonal papules that coalesce into thickened hyperkeratotic plaques. Comedo‐like plugs or evenly spaced dells can be observed on the surface of the plaques. The plugs and dells may disappear with time as the lesions age, leaving a smooth, often porcelain‐white, plaque. LS has also been described as verrucous and hyperkeratotic [1]. Histologically, an immune reaction in the basal compartment of the epidermis and the epidermal/stromal interface and around the skin adnexa can initially be observed. Basal infiltrations of lymphocytes along with a grossly vacuolated or thickened basement membrane are the diagnostic features of the initial and presclerotic stages. The initial band of inflammation shifts gradually downward, from the epidermal interface into the dermis, destroying the vascular channels and appendages and resulting in excessive deposition of altered extracellular matrix. At a late stage, scant cellular infiltrates with loss of adnexal, vascular stricture, and hyalinization and fibrosis of the dermis are observed [4]. The EuroGuiderm guideline for LS summarizes the histopathological findings as follows: compact orthohyperkeratosis, epidermal atrophy, basal cell degeneration, dermal hyalinization, and interphase dermatitis with a band‐like lymphocytic infiltrate, typically underneath the hyalinized, edematous dermis [2]. In children, histopathologically well‐developed LS lesions show an atrophic epidermis, hyperkeratosis, edema in the papillary dermis with collagen homogenization, and an underlying lymphocytic infiltrate [5]. However, a general histopathological classification for male pediatric LS patients has not yet been established.

LS is an established risk factor for malignancy and other diseases [3, 6]. In the Swedish register study, the odds ratios were increased for penile cancer (OR = 8.9), vulvar cancer (OR = 8.3), breast cancer (OR = 1.4), testicular cancer (OR = 1.4), prostate cancer (OR = 1.2), bladder cancer (OR = 1.1), leukoplakia of the vulva (OR = 253.5), and leukoplakia of the penis (OR = 5.1). In addition, in a smaller Swedish cohort from the Jönköping region, LS was also associated with Crohn's disease (OR = 2.0) and diabetes mellitus type 1 (OR = 1.9). Overall, patients with LS suffer from inflammation, itching, and pain and may harbor an increased risk for malignancies and other diseases.

The diagnosis of LS depends strictly on clinical presentation, and biomarkers for the early detection of the disease are not clinically available [7]. However, LS is associated with several immune/autoimmune and genetic targets [8]. Dysfunction of extracellular matrix protein 1 (*ECM*1), detected at the dermal–epidermis junction, has been suggested to be involved in the pathogenesis of LS [8]. ECM1 acts as a scaffold for multiple extracellular components, for example, perlecan, MMP9, and collagens. Disruption of this ECM1 scaffold may result in pathology. ECM1 expression was also significantly reduced in pediatric male LS samples [9].

There are several studies at the molecular level that analyze LS tissue for changes in RNA (mRNA or miRNA) or protein levels in comparison with normal tissue [8, 10]. Wang et al. performed transcriptome profiling and network analysis for vulvar LS, identifying mostly upregulation of T‐cell activation‐associated genes and downregulation of cell cycle progression genes [11]. Recently, single‐cell and spatial transcriptomics of vulvar LS revealed multicompartmental alterations in gene expression and signaling cross‐talk [10]. These findings reveal unifying molecular changes across keratinocytes, fibroblasts, immune cells, and melanocytes in LS tissue that can be summarized as cellular stress and damage in fibroblasts/keratinocytes; enhanced T‐cell activation and cytotoxicity; aberrant cell‒cell signaling; and increased activation of the IFN, JAK/STAT, and p53 pathways in specific cell types [10]. Profiling of microRNAs for adult LS has been performed for vulvar LS [11, 12, 13] and male LS urethral stricture disease [14]. Interestingly, all the articles revealed that the inflammation‐associated miRNA miR‐155 was predominantly overexpressed in LS tissue compared with normal tissue. However, studies on miRNAs in male pediatric LS patients have not been performed to date.

## 2 Methods

### 2.1 Patients and Tissues

Male pediatric LS patients were identified, and archived tissue samples of LS patients and non‐LS patients with routine surgical intervention with circumcision were collected from the archive of the Institute of Pathology at the University Hospital Erlangen. The age at operation ranged from 3 to 17 years (median 9 years). Hematoxylin and eosin (HE)‐stained tissue sections were reviewed by an experienced uropathologist (AH). LS and normal tissues from the same patient were marked on HE‐stained slides, and normal tissues from non‐LS male pediatric patients were marked on HE‐stained slides and macrodissected from formalin‐fixed paraffin‐embedded (FFPE) slides. The study was conducted according to the guidelines of the Declaration of Helsinki. Approval was obtained from the Ethics Committee of the Friedrich‐Alexander‐Universität Erlangen‐Nürnberg (No. 23‐206‐Br).

### 2.2 MiRNA qRT–PCR

RNA isolation from FFPE tissue was performed with the RNA RSC FFPE Kit according to the manufacturer's instructions (Promega, Madison, WI, USA). The quantification of miRNAs was conducted via a two‐step reaction using miRCURY universal reverse transcription reagents and LNA‐modified miRNA‐specific primers (Qiagen, Hilden, Germany) according to the manufacturer's instructions. Briefly, RNA (20 ng) was reverse transcribed using the miRCURY universal cDNA synthesis kit (Qiagen) and further used according to the manufacturer's recommendations. The quantitative PCRs were performed using the QuantStudio 3 real‐time PCR system (Applied Biosystems, Foster City, CA, USA) with LNA‐modified sequence‐specific primer sets and miRCURY LNA SYBR Green PCR Kits. The following miRNAs were amplified: hsa‐miR‐30b‐5p (YP00204765), hsa‐miR‐146a‐5p (YP00204688), hsa‐miR‐146b‐5p (YP02119310), hsa‐miR‐150‐5p (YP00204660), hsa‐miR‐155‐5p (YP02119311), hsa‐miR‐199b‐5p (YP00204152), hsa‐miR‐200b‐3p (YP00206071), hsa‐miR‐424‐5p (YP00204736), and hsa‐miR‐455‐5p (YP00204363). In addition, U6 snRNA (v2) (YP02119464) and SNORD44 (hsa) (YP00203902) (Qiagen) served as reference RNAs. All reactions were measured in triplicate in a final volume of 10 µL. The thermal cycling conditions were chosen according to the manufacturer's recommendations. For relative quantification, every sample was analyzed in parallel for the expression of specific miRNAs and the endogenous reference RNAs U6 snRNA and SNORD44. The relative miRNA expression levels, normalized to the reference RNAs, were calculated using the DCt method [15]. To construct the data, we applied the 40‐delta Ct method as previously described [16].

### 2.3 Statistical Analyses

The statistical analyses were performed using SPSS 28.0.0.0 (IBM, Armonk, NY, USA). The relationships between histology and inflammation and between inflammation and the age of LS patients were calculated using the Pearson chi‐squared test. Correlations between miRNA expression and histomorphological features were analyzed using Spearman's Rho test. Nonparametric tests, such as the Mann‒Whitney *U*‐test and Wilcoxon test, were applied to determine the differences in miRNA expression between the different tissues, histomorphology, and inflammation groups. To discriminate between the tumor and normal samples, we applied binary logistic regression models. To calculate the receiver–operator characteristics (ROC analysis), we used the probability function generated by the binary logistic regression algorithm. All the statistical tests were performed as two‐sided tests, and *p* values <0.05 were considered statistically significant.

## 3 Results

Ninety‐eight cases of pediatric circumcision were included in the study. In all the cases, a histological re‐examination of the foreskin specimens was performed, and 41 cases of LS were histologically confirmed. Because of the clinical suspicion of LS, 22 of the 41 patients with LS had previously received local therapy with corticosteroids. All 41 tissue samples from LS patients were re‐evaluated, and a histomorphological classification of LS was obtained by an experienced uro‐pathologist (AH). During the re‐evaluation of the LS tissue samples, areas with LS and areas with normal tissue histology (without LS) of the same sample were marked for miRNA extraction. In four LS cases, no area of normal tissue histology could be found in the samples. Overall, miRNA extraction and analysis were possible in forty samples with LS and in all 37 adjacent normal tissues. Among the 57 cases in which LS was histologically excluded (normal tissue from male pediatric non‐LS patients), miRNA extraction and analysis were possible in 51 samples. The reason for the reduced number of cases studied for miRNA expression was that either insufficient tissue was available or that insufficient amounts of RNA could be isolated.

### 3.1 Histopathological Classification

There is no histomorphological classification for male pediatric LS. In the present study, we investigated the general histomorphological criteria applied for adult LS [2]. We considered the extent and type of inflammation and lymphocytic infiltration/depletion, extent of keratosis, hyalinization/hyalinosis, sclerosis, hypoplasia of the epidermis, and presence of basal vacuoles. Finally, we propose four histomorphological groups:

- Group 1: Lichenoid basal inflammation with frequent basal vacuoles, no or only very limited hyperkeratosis, no or very limited sclerosis, and no or only weak hyalinosis
- Group 2: Dominant band‐like lymphocytic infiltration, hyalinization/hyalinosis, hyperkeratosis, atrophy of the epidermis, and often retained basal vacuoles
- Group 3: Mixed (lichenoid and band‐like) lymphocytic inflammation and extensive hyalinization/hyalinosis
- Group 4: Lymphocytic depletion (burnt out), atrophy of the epithelium, and fibrosis of the papillary sublayer of the dermis

**FIGURE 1:**
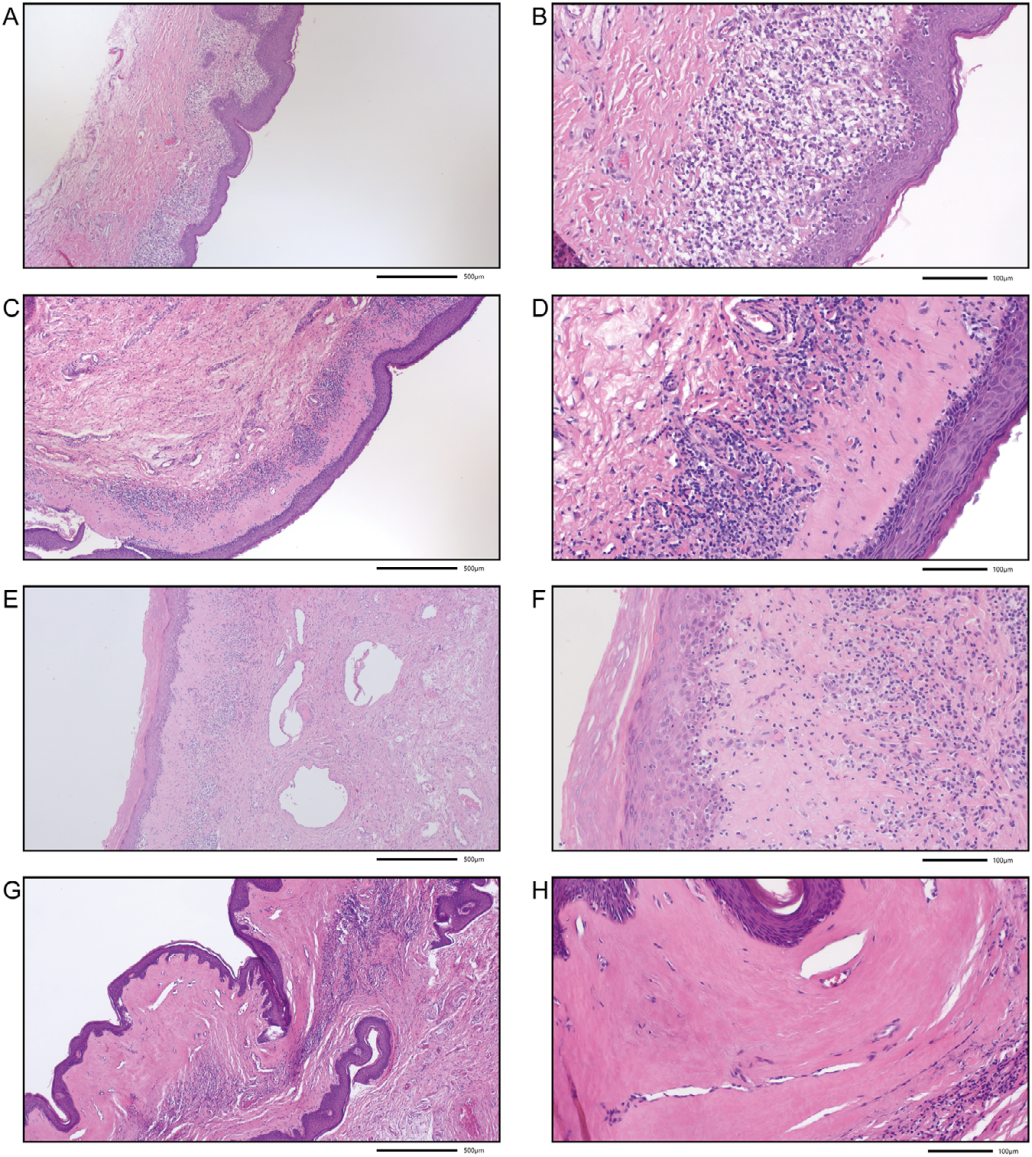
Histomorphological classification of male pediatric patients with lichen sclerosus (LS). (A and B) Group 1 with lichenoid basal inflammation with frequent basal vacuoles, no or only very limited hyperkeratosis, no or very limited sclerosis, and no or only weak hyalinosis; (C and D) Group 2 with dominant band‐like lymphocytic infiltration, hyalinization/hyalinosis, hyperkeratosis, atrophy of the epidermis, and often retained basal vacuoles; (E and F) Group 3 with mixed (lichenoid and band‐like) lymphocytic inflammation and extensive hyalinization/hyalinosis; (G and H) Group 4 with lymphocytic depletion (burnt out), atrophy of the epithelium, and fibrosis of the papillary sublayer of the dermis. The final magnification is 50× (A, C, E, G) and 200× (B, D, F, H); scale bars represent 500 and 100 µm, respectively.

Representative images for each histomorphological group are presented in Figure 1. We believe that these morphological features characterize different stages of morphological LS progression. In addition, we considered the grade of inflammation and categorized the LS samples into (i) weak, (ii) moderate, or (iii) strong inflammation.

More than half of the LS patients, that is, 21 patients, belonged to Group 2 with band‐like lymphocytic infiltration, followed by 13 patients in Group 1 with lichenoid inflammation. In contrast, only 4 patients in Group 4 exhibited lymphocytic depletion, and 3 patients in Group 3 exhibited with mixed lichenoid and band‐like lymphocytic inflammation (Table 1). The classification according to the grade of inflammation yielded 11 cases in the weak category, 14 cases in the moderate category, and 16 cases in the strong category. As expected, we observed moderate and strong inflammation in most patients in the two groups with lichenoid inflammation and band‐like lymphocytic infiltration. In contrast, in the lymphocytic depletion group, the inflammation was weak or even absent (burnt out phenotype) (*p* = 0.019; Table 2).

**TABLE 1:** Histopathological classification of male pediatric patients with lichen sclerosus.

	LS cases
**All**	41
**Histology**	
Group 1: Lichenoid inflammation	13 (31.7%)
Group 2: Band‐like lymphocytic infiltration	21 (51.2%)
Group 3: Mixed lymphocytic inflammation	3 (7.3%)
Group 4: Lymphocytic depletion	4 (9.8%)
**Grade of inflammation**	
Weak	11 (26.8%)
Moderate	14 (34.1%)
Strong	16 (39.0%)

**TABLE 2:** Cross table: relationships between histology and inflammation.

		Grade of inflammation			
		Weak	Moderate	Strong	Total
**Histology**	Group 1: Lichenoid inflammation	4	3	6	13
	Group 2: Band‐like lymphocytic infiltration	2	10	9	21
	Group 3: Mixed lymphocytic inflammation	1	1	1	3
	Group 4: Lymphocytic depletion	4	0	0	4
**Total**		11	14	16	41

*Note: p* = 0.019 Pearson chi‐squared test.

We then separated the pediatric male LS patients into two age groups based on the median age of 9 years (≤9 years vs. >9 years). We did not observe differences in histomorphology between the two age groups. However, in the younger group, more patients (*N* = 10/47.6% of patients in this group) experienced strong inflammation. In the older group, more patients (*N* = 11/55.0% of patients in this group) experienced moderate inflammation (*p* = 0.020; Table 3).

**TABLE 3:** Cross table: relationships between inflammation and the age of lichen sclerosus (LS) patients.

	Grade of inflammation			Total
**Age group**	Weak	Moderate	Strong	
**≤9 years**	8	3	10	21
**>9 years**	3	11	6	20
**Total**	11	14	16	41

*Note: p* = 0.020 Pearson chi‐squared test.

### 3.2 Expression of microRNAs in LS, Corresponding Non‐LS, and Control Tissues From Non‐LS Patients

**FIGURE 2:**
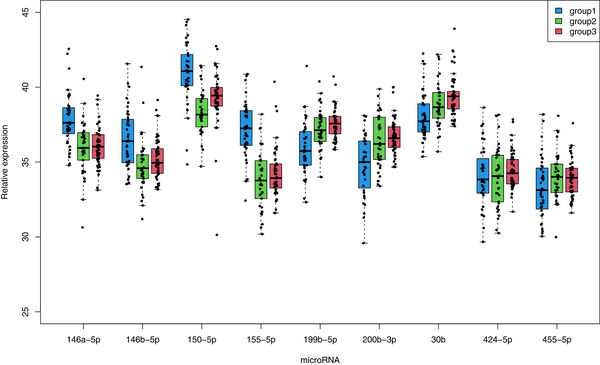
MiRNA expression in LS tissues of LS patients (Group 1), normal tissues of LS patients (Group 2), and male pediatric non‐LS patients (Group 3). The microRNAs hsa‐miR‐146a‐5p, hsa‐miR‐146b‐5p, hsa‐miR‐150‐5p, and hsa‐miR‐155‐5p were significantly upregulated in the LS tissue (Group 1; blue color) in comparison to normal tissue from these LS patients (Group 2; green color) as well as in comparison to normal tissue from other male pediatric non‐LS patients (Group 3; red color). The microRNAs hsa‐miR‐199b‐5p and hsa‐miR‐200b‐3p were significantly downregulated in LS tissue (Group 1) compared with normal tissue from LS patients (Group 2) and the control tissues from male pediatric non‐LS patients (Group 3). The microRNA hsa‐miR‐30b was significantly downregulated in LS tissue (Group 1) compared with that in non‐LS tissue from male pediatric patients (Group 2). But the microRNAs hsa‐miR‐424‐5p and hsa‐miR‐455 showed no differences in expression in any comparison.

On the basis of the literature for adult male LS, female LS, and penile cancer, we selected five microRNAs that are overexpressed in LS tissue, that is, hsa‐miR‐146a‐5p, hsa‐miR‐146b‐5p, hsa‐miR‐150‐5p, hsa‐miR‐155‐5p, and hsa‐miR‐424‐5p, and four microRNAs that are downregulated in LS, that is, hsa‐miR‐30b‐5p, hsa‐miR‐199b‐5p, hsa‐miR‐200b‐3p, and hsa‐miR‐455. We measured the expression of these microRNAs via qRT‒PCR in LS tissue, normal tissue from the corresponding LS patient, and normal tissue from male pediatric non‐LS patients. The miRNA expression levels are shown in Figure 2.

We found that hsa‐miR‐146a‐5p, hsa‐miR‐146b‐5p, hsa‐miR‐150‐5p, and hsa‐miR‐155‐5p (all *p* < 0.001, ANOVA with Tukey‐HSD post hoc test; Table S1) were significantly upregulated in the LS tissue in comparison to normal tissue from these LS patients as well as in comparison to normal tissue from other male pediatric non‐LS patients. In addition, hsa‐miR‐199b‐5p and hsa‐miR‐200b‐3p (all *p* < 0.001) were significantly downregulated in LS tissue compared with normal tissue from LS patients and the control tissues from male pediatric non‐LS patients. Hsa‐miR‐30b was significantly downregulated in LS tissue compared with that in non‐LS tissue from male pediatric patients (*p* < 0.001). However, hsa‐miR‐424‐5p and hsa‐miR‐455 showed no differences in expression in any comparison.

Next, we were interested in the correlations of the studied microRNAs separately in the three different tissue groups. All significant correlations in any tissue were positive (Spearman‐rho test; Table S2). Out of the 36 possible miRNA correlations in each tissue, after Bonferroni correction, we identified 30 significant miRNA correlations in the male pediatric non‐LS patients, 24 significant miRNA correlations in the normal tissues of the LS patients, and 16 significant miRNA correlations in the LS tissues of the LS patients (Table S2). Taken together, these findings indicate that the number of miRNA correlations is clearly lower in LS tissue than in the two normal tissues. In all the cases, the miRNA expression levels in the corresponding normal tissues of the LS patients were rather comparable to those of the male pediatric non‐LS patients but not to those of the LS patients.

The area under the curve (AUC) for distinguishing between LS tissue and normal tissue from LS patient tissue based on miRNA expression levels yielded the following values: hsa‐miR‐155‐5p (AUC = 0.892) > hsa‐miR‐150‐5p (0.864) > hsa‐miR‐146a‐5p (0.785) > hsa‐miR‐146b‐5p (0.751) > hsa‐miR‐199b‐5p (0.736) > hsa‐miR‐200b‐3p (0.713) > hsa‐miR‐30b‐5p (0.676) > hsa‐miR‐455‐5p (0.622) > hsa‐miR‐424‐5p (0.526). Considering both hsa‐miR‐155 and hsa‐miR‐150 together, an AUC of 0.906 was obtained.

The AUC for distinguishing between LS tissue and normal tissue from male pediatric non‐LS patients based on miRNA expression levels yielded the following values: hsa‐miR‐155‐5p (AUC = 0.882) > hsa‐miR‐150‐5p (0.811) > hsa‐miR‐146a‐5p (0.789) > hsa‐miR‐199b‐5p (0.778) > hsa‐miR‐200b‐3p (0.753) > hsa‐miR‐30b‐5p (0.732) > hsa‐miR‐146b‐5p (0.726) > hsa‐miR‐455‐5p (0.622) > hsa‐miR‐424‐5p (0.517). Considering hsa‐miR‐155 and hsa‐miR‐150 together, an AUC of 0.874 was obtained. Here, hsa‐miR‐155‐5p was the single miRNA with the highest AUC value.

Next, we studied whether miRNA expression differed between the histological groups and inflammation categories. Only hsa‐miR‐424‐5p was significantly lower in Group 3 (mixed lymphocytic inflammation) than in Group 1 (lichenoid inflammation) (*p* = 0.025). In the inflammation grade categories, hsa‐miR‐146a‐5p was significantly higher in category 2 (moderate) than in category 1 (weak) (*p* = 0.026). Hsa‐miR‐199b‐5p was more highly expressed in categories 2 (moderate) to 3 (strong) (*p* = 0.017).

When the pediatric male LS patients were separated into two age groups (≤9 years vs. >9 years), the expression of the miRNA hsa‐miR‐150‐5p (*p* = 0.049) was significantly higher in the younger age group. This result is consistent with the finding that this miRNA is generally expressed at higher levels in LS tissue than in normal tissue from LS patients.

**FIGURE 3:**
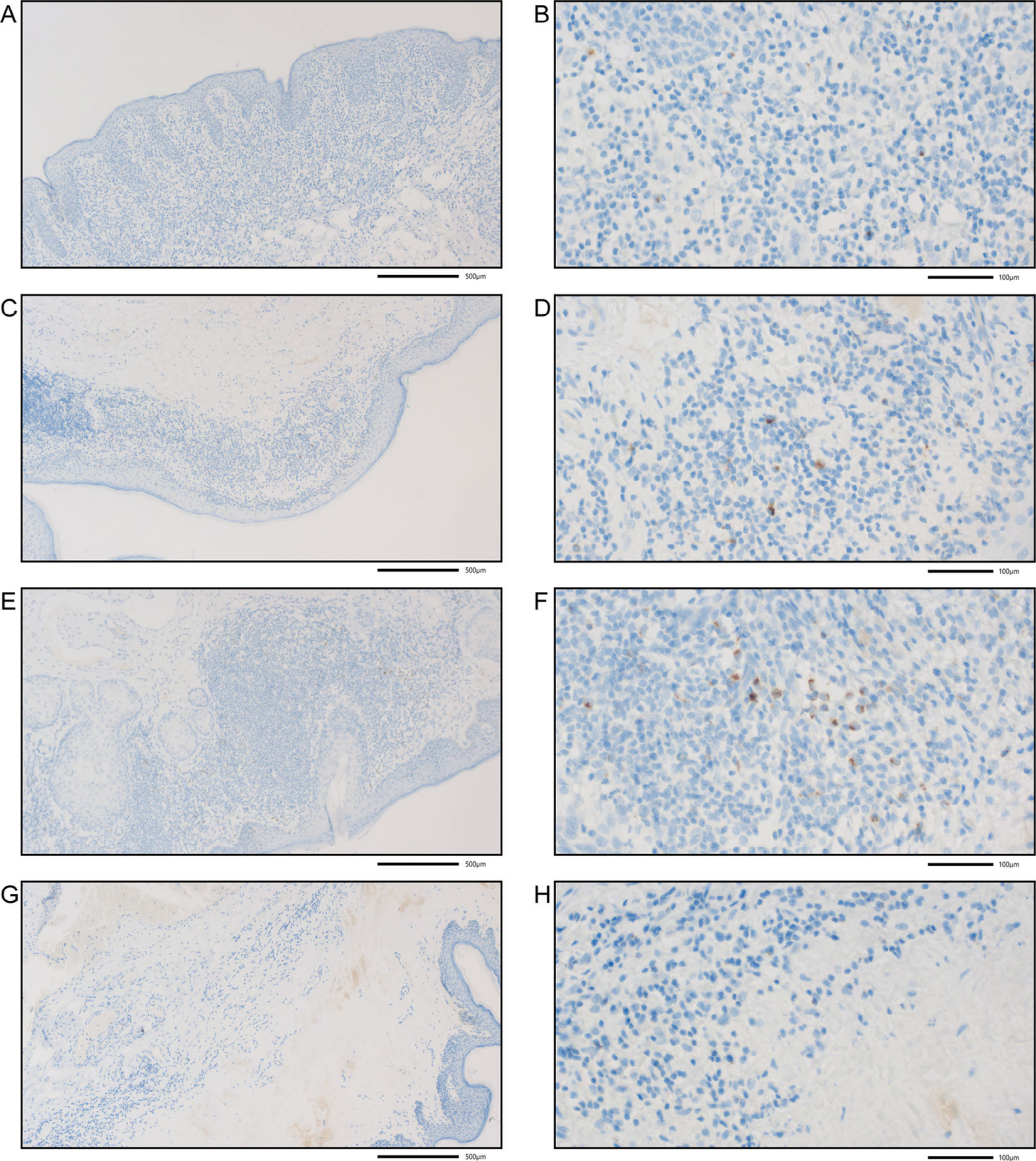
Immunohistochemical staining for granzyme B in the four histomorphological groups of male pediatric patients with LS. Granzyme B staining concerns single lymphocytes that represent cytotoxic T cells and NK cells. (A and B) Group 1 (lichenoid basal inflammation); (C and D) Group 2 (band‐like lymphocytic infiltration); (E and F) Group 3 (mixed lichenoid and band‐like lymphocytic inflammation); (G and H) Group 4 (lymphocytic depletion/burnt out). The final magnification is 50× (A, C, E, G) and 400× (B, D, F, H); scale bars represent 500 and 100 µm, respectively.

As an exploratory approach, we tested whether we could detect by immunohistochemistry a selected protein known to be correlated to inflammation; we chose the protein granzyme B. Granzyme B is a well‐known inflammation marker. The expression of granzyme B in the immune cells of LS tissue was gradually decreasing in the four histomorphological groups: Group 3 > Group 2 > Group 1 > Group 4 (Figure 3). The strongest expression of granzyme B was detected in the LS tissue of Group 3 (mixed lichenoid and band‐like lymphocytic inflammation) and no expression in Group 4 (lymphocytic depletion), and in no case has a granzyme B staining been detected in the corresponding normal tissue, as expected.

## 4 Discussion

Until recently, there has been no generally applied histomorphological classification and no study of miRNA expression in male pediatric LS patients. Considering the LS classification for adult male patients [2], we categorized pediatric LS patients into four groups according to their histomorphology: (i) Group 1: lichenoid inflammation; (ii) Group 2: band‐like lymphocytic infiltration; (iii) Group 3: mixed lymphocytic inflammation; or (iv) Group 4: lymphocytic depletion. In addition, patients were also classified according to the grade of inflammation: (i) weak, (ii) moderate, or (iii) strong inflammation. The majority of the patients had lichenoid inflammation and band‐like lymphocytic infiltration. Accordingly, most patients with moderate or strong inflammation were in these two groups. However, in the lymphocytic depletion group, the inflammation was very weak or even burnt out. It will be interesting to investigate the different histopathological groups using large clinical cohorts of pediatric LS patients to correlate these groups with clinical outcomes.

We tested whether microRNA expression in LS tissue or normal tissue from LS patients was affected by previous corticosteroid treatment (*N* = 22 out of 41). This was not the case (data not shown). Our microRNA analysis compared the expression levels of nine selected microRNAs known to be dysregulated in adult LS tissues. Here, we analyzed the expression levels in the LS tissues and corresponding normal tissues of pediatric male LS patients and in the normal tissues of non‐LS patients. We found that hsa‐miR‐146a‐5p, hsa‐miR‐146b‐5p, hsa‐miR‐150‐5p, and hsa‐miR‐155‐5p were significantly upregulated in the LS tissue compared with normal tissue from these LS patients as well as normal tissue from male pediatric non‐LS patients. The miRNAs hsa‐miR‐146a‐5p, hsa‐miR‐146b‐5p and hsa‐miR‐150‐5pare upregulated in rheumatoid arthritis [17] and penile cancer [18] but not in vulvar cancer [19]. However, although hsa‐miR‐146a‐5p and hsa‐miR‐155‐5p affect various essential immune functions, these two miRNAs are suggested to act in opposite ways [20]. In detail, hsa‐miR‐155‐5p and hsa‐miR‐150‐5p have been recently reported to be upregulated in HPV‐associated penile carcinoma [21] but not yet in lichen‐associated penile carcinoma. In addition, the upregulation of both miRNAs is associated with resistance to anticancer drugs (doxorubicin, cisplatin, paclitaxel, gefitinib, and taxanes) in penile cancer [21]. Hsa‐miR‐199b‐5p and hsa‐miR‐200b‐3p were significantly downregulated in LS tissue compared with the corresponding normal tissues of LS patients and male pediatric non‐LS patients. Hsa‐miR‐30b was significantly downregulated in LS tissue compared with that in male pediatric non‐LS patients. The miRNAs hsa‐miR‐30b‐5p, hsa‐miR‐199b‐5p, and hsa‐miR‐200b‐3p have also been reported to be downregulated in penile cancer [18] but not in rheumatoid arthritis [17] or vulvar cancer [19].

Correlations of the nine microRNAs separately analyzed in the three different tissues revealed that the number of miRNA correlations clearly decreased in LS tissue compared with the two normal tissues. This may indicate a switch from a physiological status to a pathological status.

However, together, the high number of miRNA correlations suggests either general regulation, for example, by transcription factors, or that the miRNAs influence each other directly or indirectly by suppressing regulators of miRNA expression [22] given that all miRNA correlations in all tissues are positive. Hill and Tran described three types of miRNA interactions: (i) direct miRNA–miRNA interactions when an miRNA binds another miRNA in a complementary fashion; (ii) miRNA can modulate the expression of another miRNA by controlling its transcription or regulatory pathways as part of a gene regulatory network; and (iii) miRNAs can regulate the expression of miRNA biogenesis pathway components, which affect the production of several miRNAs [22]. These relationships have already been described among transcription factors, miRNAs, and DNA methylation in several autoimmune and inflammatory diseases [23] but are also the subject of current research. However, these regulatory pathways generally remain uncharacterized for LS and need further investigation.

A gene expression study in boys with phimosis and LS and in an age‐matched group of boys with phimosis but no LS revealed distinct expression patterns of tissue remodeling‐associated genes in LS. However, none of the upregulated genes were targets of our downregulated miRNAs [24].

In our study, it was possible to distinguish between LS tissue and normal tissue from an LS patient based on miRNA expression levels, with an AUC of >0.81 for each of the two microRNAs, hsa‐miR‐155‐5p and hsa‐miR‐150‐5p. These results suggest that these two microRNAs are diagnostic and possibly therapeutic targets for male pediatric LS. In addition, distinguishing between LS tissue and normal tissue from male pediatric non‐LS patients was already possible using hsa‐miR‐155.

Both miRNAs are involved in regulatory processes of various inflammatory and autoimmune diseases, including multiple sclerosis, sepsis, rheumatoid arthritis, Sjögren's syndrome, and systemic lupus erythematosus [17, 25, 26, 27, 28]. In addition, miR‐155 upregulation has also been reported in inflammatory bowel disease, ulcerative colitis, Crohn's disease, type 1 diabetes, systemic sclerosis, and atopic dermatitis, reviewed in [29, 30]. Hsa‐miR‐155 detected in serum/plasma has been reported as a diagnostic marker for sepsis, with an AUC of 0.85 [31]. Overall, hsa‐miR‐150 suppresses anti‐inflammatory pathways, and miR‐155 activates proinflammatory pathways [28].

Hsa‐miR‐150 controls B‐ and T‐cell differentiation and is expressed in mature B cells and T cells [32]. In particular, hsa‐miR‐150 is expressed in invariant natural killer T (iNKT) cells, and its expression is gradually upregulated during iNKT cell maturation [33].

Hsa‐miR150 has the potential target genes encoding interleukin (IL)‐6, nuclear factor (κB)‐1, Janus kinase (JAK)‐2, IL 1 receptor‐associated kinase (IRAK)‐2, c‐MYB, and the tumor suppressor P53 [34, 35, 36, 37, 38, 39].

Hsa‐miR‐155 is expressed and functions in a variety of immune cell types, such as monocytes, macrophages, dendritic cells, natural killer (NK) lymphocytes, various T‐cell subsets, and B lymphocytes [20, 29, 40]. Hsa‐miR‐155 enhances regulatory T cells (Tregs) and inflammatory T helper (Th) 1 and 17 cell differentiation [41]. Hsa‐miR‐155‐5p drives the inflammatory activation of macrophages and monocytes by targeting inhibitors of the TLR and cytokine receptor pathways, thus resulting in increased production of the cytokines TNF, IL‐6, IL‐8, and IL‐1β [42].

As a target gene, hsa‐miR‐155 is a suppressor of cytokine signaling (SOCS: SOCS1, SOCS5, SOCS6) that inhibits the JAK/STAT pathway in addition to different cytokines [40, 43, 44, 45, 46, 47]. Furthermore, hsa‐miR‐155 inhibits the transcription factor forkhead box O3 (FOXO3), the transcription repressor BCL6, and the cell cycle regulator cyclin‐dependent kinase inhibitor 1B (CDKN1B/p27) [12, 48, 49]. Hsa‐miR‐155 activates the TLR/MyD88, NF‐κB, JNK/STAT, PI3K/Akt, and MAPK signaling pathways and regulates the Wnt/β‐catenin signaling pathway reviewed in [29].

Until recently, a therapeutic approach to inhibit hsa‐miR‐150‐5p has not been available. Inhibition of hsa‐miR‐155‐5p by a commercially available anti‐miR‐155 locked nucleic acid‐antisense oligonucleotide (MRG‐106/cobomarsen; Miragen Therapeutics name changed 2021 to Viridian Therapeutics, Boulder, Colorado, USA) has been described. In 2017, the FDA and the European Medicines Agency (EMA) granted orphan drug designation to cobomarsen for the treatment of mycosis fungoides‐type cutaneous T‐cell lymphoma (CTCL). Cobomarsen has been used in two clinical trials, the SOLAR trial (NCT03713320) and a subsequent phase II trial called PRISM (NCT03837457), both of which treated MF‐CTCL patients. Treatment with cobomarsen effectively promoted sustained improvements in CAILS, mSWAT, and Skindex‐29, which are measures of lesion burden and quality of life, respectively, in MF‐CTLC patients [50]. In addition, no serious adverse events attributed to cobomarsen and no evidence of immunosuppression were observed [50]. However, the company Miragen decided to discontinue further internal development of cobomarsen based on reasons unrelated to safety and efficacy [51]. Cobomarsen was withdrawn from the FDA on 02/25/2022 and from the Union Register (EMA) of orphan medicinal products in June 2022 on request of the Sponsor https://www.accessdata.fda.gov/scripts/opdlisting/oopd/detailedIndex.cfm?cfgridkey=748520 (accessed: August 12, 2025); https://www.ema.europa.eu/en/medicines/human/orphan‐designations/eu‐3‐17‐1872 (accessed: August 12, 2025). However, the use of cobomarsen in autoimmune and/or inflammatory diseases has not yet been studied.

An inhibitor of hsa‐miR‐155, that is, the nonsteroidal anti‐inflammatory drug β‐d‐mannuronic acid (M2000), was tested in a clinical phase III study in rheumatoid arthritis patients (IRCT2017100213739N10). The authors reported that hsa‐miR‐155 gene expression levels significantly decreased in the PBMCs of RA patients treated with M2000 after 12 weeks compared with baseline [52, 53].

Vildagliptin, a dipeptidyl peptidase‐4 (DPP4) inhibitor, has been reported to reduce inflammation, inhibit the PI3K/Akt/NFκB pathway, and attenuate the expression of hsa‐miR‐146a in ulcerative colitis mouse model [54]. Vildagliptin is an EMA‐ and FDA‐approved treatment for patients with type 2 diabetes mellitus [55]. Recently, a phase III study (NCT06348706) with vildagliptin was performed in patients with diabetes mellitus type 1 and nonalcoholic steatohepatitis. It improved glycemic control, dyslipidemia, and matrix metalloprotease 14 levels and decreased liver stiffness and carotid intima media thickness. On the basis of these activities, the treatment reduced subclinical atherosclerosis and disease progression [56]. In the abovementioned study by Gulin et al., LS was associated with a 1.9‐fold increased OR for diabetes mellitus type 1 [6]; thus, a DPP4 inhibitor could represent a potential candidate for future studies on LS treatments.

Although LS can appear at any age, the incidence of LS has a typical bimodal onset peak in prepubertal children and postmenopausal women [57]. The timing of puberty in boys is generally between 9 and 14 years. Interestingly, when the pediatric male LS patients in our study were separated into two age groups (≤9 years vs. >9 years), the prepubertal group presented a higher grade of inflammation (*p* = 0.020). Additionally, the miRNA hsa‐miR‐150‐5p was significantly more highly expressed in this group than in the older cohort (*p* = 0.049). Although a significant influence of hormones on the pathogenesis of LS is suspected, their exact role remains unclear, and the effectiveness of hormone therapies in LS patients remains a matter of debate [7]. Hsa‐miR‐150 regulates steroidogenesis in mouse testicular Leydig cells [58]. However, further studies are needed to elucidate the mechanisms linking miRNA expression, hormonal activity, and inflammation in the context of LS, potentially paving the way for novel therapeutic strategies.

To test whether the inflammation in the LS tissue is represented also by the protein expression of immune cells, we chose granzyme B. Granzyme B is well known as an inflammation marker that is primarily expressed by cytotoxic T lymphocytes and NK cells [59, 60]. In addition, granzyme B is negatively regulated by hsa‐miR‐199b‐5p [61]. As we detected a downregulation of hsa‐miR‐199b‐5p in the LS tissues in comparison to the normal tissue from LS patients, we expected a higher expression of granzyme B in the LS tissue in comparison to the normal tissue from LS patients. The expression of granzyme B in the immune cells of LS tissue was gradually decreasing in the four histomorphological groups: Group 3 (strongest) > Group 2 > Group 1 > Group 4 (negative), and additionally, no granzyme B expression was detected in any corresponding normal tissue. The strongest expression of granzyme B in the LS tissue of Group 3 (mixed lichenoid and band‐like lymphocytic inflammation) was expected, as this histomorphological group shows the strongest extent of inflammation and the features of Groups 1 + 2 together; moreover, in Group 4 with lymphocytic depletion, the inflammation is burnt out, and therefore, no granzyme B expression was expected. Furthermore, as granzyme B is an inflammation marker, it was as expected not present in the corresponding normal tissue.

Our study has limitations. It is a single‐center study with limited male pediatric patients with LS. Therefore, the number of patients in the new established pathomorphological groups and its statistical analysis are also limited. Because of the restricted sample size of macrodissected LS tissue, the study was only possible for a selected number of microRNAs. Altogether, our study has to be followed up and complemented by a multicenter study that should also include further microRNAs.

In summary, we characterized four histomorphologically different groups and three different grades of inflammation observed in male pediatric LS patients. Four microRNAs (hsa‐miR‐146a‐5p, hsa‐miR‐146b‐5p, hsa‐miR‐150‐5p, and hsa‐miR‐155‐5p) were significantly upregulated, and two miRNAs (hsa‐miR‐199b‐5p and hsa‐miR‐200b‐3p) were significantly downregulated in LS tissue compared with adjacent normal tissue as well as normal tissue from male pediatric non‐LS patients. We suggest that these miRNAs are diagnostic markers for LS in pediatric male patients and that they may represent therapeutic targets in the future.

## Author Contributions

**Marios Marcou**: conceptualization, data curation, formal analysis, funding acquisition, project administration, supervision, writing – original draft preparation, writing – review and editing. **Helge Taubert**: conceptualization, data curation, formal analysis, funding acquisition, project administration, supervision, validation, visualization, writing – original draft preparation, writing – review and editing. **Sven Wach**: conceptualization, data curation, formal analysis, methodology, project administration, supervision, validation, visualization, writing – original draft preparation, writing – review and editing. **Arndt Hartmann**: data curation, methodology, project administration, resources, supervision, visualization, writing – review and editing. **Robert Stöhr**: data curation, investigation, resources, writing – review and editing. **Valerie Flammang**: data curation, investigation, validation, writing – review and editing. **Katrin Weigelt**: data curation, investigation, validation, writing – review and editing. **Bernd Wullich**: project administration, supervision, writing – review and editing. **Carol Geppert**: resources, visualization, writing – review and editing. **Frederik A. Stuebs**: resources, writing – review and editing. **Matthias W. Beckmann**: supervision, writing – review and editing.

## Ethics Statement

The study has been performed according to the Declaration of Helsinki, and the procedures have been approved by the local ethics committee (23‐206‐Br).

## Consent

No written consent for publication has been obtained from the patients as there is no patient‐identifiable data included in this series.

## Conflicts of Interest

The authors declare no conflicts of interest.

## Supporting information




**Table S1**: Comparison of miRNA expressions: ANOVA with Tukey‐HSD post hoc test.
**Table S2**: MiRNA correlations (Spearman rho tests).

## Data Availability

Data are provided within the manuscript or Supporting Information files. The detailed datasets used and analyzed during the present study are available from the corresponding author upon reasonable request.
