# Supplementary material for: Histopathological Characterization and Differential Expression of miRNAs in Male Pediatric Patients With Lichen Sclerosus
Source: Andrology. 2025 Dec 17;14(3):822–32. doi: 10.1111/andr.70157 (PMC12919680; doi:10.1111/andr.70157)
Supplement: Supplementary file 1 — Table S1: Comparison of miRNA expressions: ANOVA with Tukey‐HSD post hoc test. Table S2: MiRNA correlations (Spearman rho tests). [file ANDR-14-822-s001.docx]

**Suppl. Data**

Histopathological characterization and differential expression of miRNAs in male pediatric patients with lichen sclerosus

**Valerie Flammang** **^1, #^, Arndt Hartmann ^2, 3, 4, 5, #^, Robert Stöhr ^2, 3, 4, 5^, Katrin Weigelt ^1^, Carol Geppert ^2, 3, 4, 5^, Frederik A. Stuebs ^3, 4, 5, 6^, Matthias W. Beckmann ^3, 4, 5, 6^,** **Bernd Wullich ^1, 3, 4, 5^, Helge Taubert ^1, 3, 4, 5^ *, Marios Marcou ^1, 3, 4,^** ^§^**, Sven Wach ^1, 3, 4, 5,^** ^§^

**Suppl. Table S1:** Comparison of miRNA expressions: ANOVA with Tukey-HSD post hoc test

| **depending variable** | | | | **average difference** | **Significance** | **95% confidence interval** | |
| --- | --- | --- | --- | --- | --- | --- | --- |
|  |  |  |  |  |  | lower border | upper border |
| 30b | Tukey-HSD | 1 | 2 | -0.756 | 0.065 | -1.549 | 0.037 |
|  |  |  | 3 | -1.350^*^ | **<0.001** | -2.078 | -0.622 |
|  |  | 2 | 3 | -0.594 | 0.145 | -1.339 | 0.150 |
| 146a-5p | Tukey-HSD | 1 | 2 | 1.871^*^ | **<0.001** | 1.000 | 2.744 |
|  |  |  | 3 | 1.769^*^ | **<0.001** | 0.963 | 2.577 |
|  |  | 2 | 3 | -0.102 | 0.954 | -0.927 | 0.724 |
| 146b-5p | Tukey-HSD | 1 | 2 | 1.736^*^ | **<0.001** | 0.809 | 2.664 |
|  |  |  | 3 | 1.359^*^ | **0.001** | 0.501 | 2.219 |
|  |  | 2 | 3 | -0.376 | 0.568 | -1.254 | 0.502 |
| 150-5p | Tukey-HSD | 1 | 2 | 2.616^*^ | **<0.001** | 1.636 | 3.597 |
|  |  |  | 3 | 1.624^*^ | **<0.001** | 0.717 | 2.532 |
|  |  | 2 | 3 | -.992^*^ | 0.033 | -1.920 | -0.064 |
| 155-5p | Tukey-HSD | 1 | 2 | 4.243^*^ | **<0.001** | 2.353 | 6.133 |
|  |  |  | 3 | 2.879^*^ | **<0.001** | 1.129 | 4.629 |
|  |  | 2 | 3 | -1.364 | 0.171 | -3.153 | 0.425 |
| 199b-5p | Tukey-HSD | 1 | 2 | -1.286^*^ | **<0.001** | -2.037 | -0.535 |
|  |  |  | 3 | -1.648^*^ | **<0.001** | -2.344 | -0.953 |
|  |  | 2 | 3 | -0.362 | 0.450 | -1.073 | 0.348 |
| 200b-3p | Tukey-HSD | 1 | 2 | -2.019^*^ | **0.001** | -3.267 | -0.771 |
|  |  |  | 3 | -2.324^*^ | **<0.001** | -3.480 | -1.169 |
|  |  | 2 | 3 | -0.305 | 0.814 | -1.486 | 0.876 |
| 424-5p | Tukey-HSD | 1 | 2 | 0.187 | 0.884 | -0.751 | 1.125 |
|  |  |  | 3 | -0.271 | 0.740 | -1.140 | 0.597 |
|  |  | 2 | 3 | -0.458 | 0.442 | -1.346 | 0.430 |
| 455-5p | Tukey-HSD | 1 | 2 | -1.145 | 0.061 | -2.332 | 0.042 |
|  |  |  | 3 | -0.978 | 0.092 | -2.078 | 0.121 |
|  |  | 2 | 3 | 0.167 | 0.934 | -0.957 | 1.291 |

*. The average difference is at <0.05 significant.

b. In Dunnett-T-Tests one group is considered as control group. All other groups are compared with the control group.

**bold face p** is below the Bonferroni adjusted a-level of 0.00185

**Suppl. Table S2**: miRNA correlations (Spearman Rho tests)

1. **miRNA correlations in LS tissues of LS cases**

|  | | | 30b | 146a-5p | 146b-5p | 150-5p | 155-5p | 199b-5p | 200b-3p | 424-5p | 455-5p |
| --- | --- | --- | --- | --- | --- | --- | --- | --- | --- | --- | --- |
| Spearman-Rho | 30b | correlation coefficient | 1.000 |  |  |  |  |  |  |  |  |
|  |  | Sig. (2-sided) |  |  |  |  |  |  |  |  |  |
|  |  | N | 40 |  |  |  |  |  |  |  |  |
|  | 146a-5p | correlation coefficient | .484^**^ | 1.000 |  |  |  |  |  |  |  |
|  |  | Sig. (2-sided) | **0.002** |  |  |  |  |  |  |  |  |
|  |  | N | 40 | 40 |  |  |  |  |  |  |  |
|  | 146b-5p | correlation coefficient | .659^**^ | .528^**^ | 1.000 |  |  |  |  |  |  |
|  |  | Sig. (2-sided) | **0.000** | **0.000** |  |  |  |  |  |  |  |
|  |  | N | 40 | 40 |  |  |  |  |  |  |  |
|  | 150-5p | correlation coefficient | .588^**^ | .602^**^ | .715^**^ | 1.000 |  |  |  |  |  |
|  |  | Sig. (2-sided) | **0.000** | **0.000** | **0.000** |  |  |  |  |  |  |
|  |  | N | 40 | 40 | 40 | 40 |  |  |  |  |  |
|  | 155-5p | correlation coefficient | .541^**^ | .652^**^ | .446^**^ | .745^**^ | 1.000 |  |  |  |  |
|  |  | Sig. (2-sided) | **0.000** | **0.000** | 0.004 | **0.000** |  |  |  |  |  |
|  |  | N | 40 | 40 | 40 | 40 | 40 |  |  |  |  |
|  | 199b-5p | correlation coefficient | .441^**^ | .586^**^ | 0.242 | 0.227 | .357^*^ | 1.000 |  |  |  |
|  |  | Sig. (2-sided) | 0.004 | **0.000** | 0.133 | 0.158 | 0.024 |  |  |  |  |
|  |  | N | 40 | 40 | 40 | 40 | 40 | 40 |  |  |  |
|  | 200b-3p | correlation coefficient | .559^**^ | .449^**^ | 0.295 | 0.198 | 0.232 | .411^**^ | 1.000 |  |  |
|  |  | Sig. (2-sided) | **0.000** | 0.004 | 0.064 | 0.221 | 0.151 | 0.009 |  |  |  |
|  |  | N | 40 | 40 | 40 | 40 | 40 | 40 | 40 |  |  |
|  | 424-5p | correlation coefficient | .419^**^ | 0.302 | .598^**^ | .593^**^ | .430^**^ | 0.256 | -0.081 | 1.000 |  |
|  |  | Sig. (2-sided) | **0.007** | 0.059 | **0.000** | **0.000** | 0.006 | 0.110 | 0.618 |  |  |
|  |  | N | 40 | 40 | 40 | 40 | 40 | 40 | 40 | 40 |  |
|  | 455-5p | correlation coefficient | .678^**^ | .402^*^ | .731^**^ | .569^**^ | .469^**^ | 0.248 | .392^*^ | .680^**^ | 1.000 |
|  |  | Sig. (2-sided) | **0.000** | 0.010 | **0.000** | **0.000** | 0.002 | 0.123 | 0.012 | **0.000** |  |
|  |  | N | 40 | 40 | 40 | 40 | 40 | 40 | 40 | 40 | 40 |

**. The correlation is on the 0.01 level significant (two-sided).

*. The correlation is on the 0.05 level significant (two-sided).

All values in bold font are also significant after Bonferroni correction for multiple testing

1. **miRNA correlations in normal tissue of corresponding LS cases**

|  |  | 30b | 146a-5p | 146b-5p | 150-5p | 155-5p | 199b-5p | 200b-3p | 424-5p | 455-5p |
| --- | --- | --- | --- | --- | --- | --- | --- | --- | --- | --- |
| 30b | correlation coefficient | 1.000 |  |  |  |  |  |  |  |  |
|  | Sig. (2-sided) |  |  |  |  |  |  |  |  |  |
|  | N | 37 |  |  |  |  |  |  |  |  |
| 146a-5p | correlation coefficient | .624^**^ | 1.000 |  |  |  |  |  |  |  |
|  | Sig. (2-sided) | **0.000** |  |  |  |  |  |  |  |  |
|  | N | 37 | 37 |  |  |  |  |  |  |  |
| 146b-5p | correlation coefficient | .749^**^ | .648^**^ | 1.000 |  |  |  |  |  |  |
|  | Sig. (2-sided) | **0.000** | **0.000** |  |  |  |  |  |  |  |
|  | N | 37 | 37 | 37 |  |  |  |  |  |  |
| 150-5p | correlation coefficient | .799^**^ | .616^**^ | .777^**^ | 1.000 |  |  |  |  |  |
|  | Sig. (2-sided) | **0.000** | **0.000** | **0.000** |  |  |  |  |  |  |
|  | N | 37 | 37 | 37 | 37 |  |  |  |  |  |
| 155-5p | correlation coefficient | .490^**^ | .676^**^ | .462^**^ | .660^**^ | 1.000 |  |  |  |  |
|  | Sig. (2-sided) | **0.002** | **0.000** | 0.004 | **0.000** |  |  |  |  |  |
|  | N | 37 | 37 | 37 | 37 | 37 |  |  |  |  |
| 199b-5p | correlation coefficient | .634^**^ | .759^**^ | .446^**^ | .533^**^ | .387^*^ | 1.000 |  |  |  |
|  | Sig. (2-sided) | **0.000** | **0.000** | 0.006 | **0.001** | 0.018 |  |  |  |  |
|  | N | 37 | 37 | 37 | 37 | 37 | 37 |  |  |  |
| 200b-3p | correlation coefficient | .663^**^ | .654^**^ | .524^**^ | .425^**^ | 0.310 | .600^**^ | 1.000 |  |  |
|  | Sig. (2-sided) | **0.000** | **0.000** | **0.001** | 0.009 | 0.062 | **0.000** |  |  |  |
|  | N | 37 | 37 | 37 | 37 | 37 | 37 | 37 |  |  |
| 424-5p | correlation coefficient | .537^**^ | .405^*^ | .670^**^ | .676^**^ | .403^*^ | .381^*^ | 0.104 | 1.000 |  |
|  | Sig. (2-sided) | **0.001** | 0.013 | **0.000** | **0.000** | 0.013 | 0.020 | 0.542 |  |  |
|  | N | 37 | 37 | 37 | 37 | 37 | 37 | 37 | 37 |  |
| 455-5p | correlation coefficient | .819^**^ | .517^**^ | .820^**^ | .707^**^ | .431^**^ | .522^**^ | .537^**^ | .666^**^ | 1.000 |
|  | Sig. (2-sided) | **0.000** | 0.001 | **0.000** | **0.000** | 0.008 | **0.001** | **0.001** | **0.000** |  |
|  | N | 37 | 37 | 37 | 37 | 37 | 37 | 37 | 37 | 37 |

**. The correlation is on the 0.01 level significant (two-sided).

*. The correlation is on the 0.05 level significant (two-sided).

All values in bold font are also significant after Bonferroni correction for multiple testing

1. **miRNA correlations in normal tissue of Non-LS cases**

|  |  | 30b | 146a-5p | 146b-5p | 150-5p | 155-5p | 199b-5p | 200b-3p | 424-5p | 455-5p |
| --- | --- | --- | --- | --- | --- | --- | --- | --- | --- | --- |
| 30b | correlation coefficient | 1.000 |  |  |  |  |  |  |  |  |
|  | Sig. (2-sided) |  |  |  |  |  |  |  |  |  |
|  | N | 53 |  |  |  |  |  |  |  |  |
| 146a-5p | correlation coefficient | .633^**^ | 1.000 |  |  |  |  |  |  |  |
|  | Sig. (2-sided) | **0.000** |  |  |  |  |  |  |  |  |
|  | N | 51 | 51 |  |  |  |  |  |  |  |
| 146b-5p | correlation coefficient | .545^**^ | .698^**^ | 1.000 |  |  |  |  |  |  |
|  | Sig. (2-sided) | **0.000** | **0.000** |  |  |  |  |  |  |  |
|  | N | 51 | 51 | 51 |  |  |  |  |  |  |
| 150-5p | correlation coefficient | .664^**^ | .668^**^ | .650^**^ | 1.000 |  |  |  |  |  |
|  | Sig. (2-sided) | **0.000** | **0.000** | **0.000** |  |  |  |  |  |  |
|  | N | 51 | 51 | 51 | 51 |  |  |  |  |  |
| 155-5p | correlation coefficient | .388^**^ | .544^**^ | .666^**^ | .529^**^ | 1.000 |  |  |  |  |
|  | Sig. (2-sided) | 0.005 | **0.000** | **0.000** | **0.000** |  |  |  |  |  |
|  | N | 51 | 51 | 51 | 51 | 51 |  |  |  |  |
| 199b-5p | correlation coefficient | .611^**^ | .696^**^ | .634^**^ | .532^**^ | .342^*^ | 1.000 |  |  |  |
|  | Sig. (2-sided) | **0.000** | **0.000** | **0.000** | **0.000** | 0.014 |  |  |  |  |
|  | N | 51 | 51 | 51 | 51 | 51 | 51 |  |  |  |
| 200b-3p | correlation coefficient | .704^**^ | .775^**^ | .611^**^ | .745^**^ | .392^**^ | .713^**^ | 1.000 |  |  |
|  | Sig. (2-sided) | **0.000** | **0.000** | **0.000** | **0.000** | 0.004 | **0.000** |  |  |  |
|  | N | 51 | 51 | 51 | 51 | 51 | 51 | 51 |  |  |
| 424-5p | correlation coefficient | .428^**^ | .555^**^ | .566^**^ | 0.234 | 0.217 | .808^**^ | .512^**^ | 1.000 |  |
|  | Sig. (2-sided) | 0.002 | **0.000** | **0.000** | 0.098 | 0.127 | **0.000** | **0.000** |  |  |
|  | N | 51 | 51 | 51 | 51 | 51 | 51 | 51 | 51 |  |
| 455-5p | correlation coefficient | .766^**^ | .666^**^ | .695^**^ | .686^**^ | .543^**^ | .788^**^ | .801^**^ | .600^**^ | 1.000 |
|  | Sig. (2-sided) | **0.000** | **0.000** | **0.000** | **0.000** | **0.000** | **0.000** | **0.000** | **0.000** |  |
|  | N | 51 | 51 | 51 | 51 | 51 | 51 | 51 | 51 | 51 |

**. The correlation is on the 0.01 level significant (two-sided).

*. The correlation is on the 0.05 level significant (two-sided).

All values in bold font are also significant after Bonferroni correction for multiple testing
